# Toward Continuous GPS Carrier-Phase Time Transfer: Eliminating the Time Discontinuity at an Anomaly

**DOI:** 10.6028/jres.120.017

**Published:** 2015-11-17

**Authors:** Jian Yao, Judah Levine, Marc Weiss

**Affiliations:** 1 National Institute of Standards and Technology, Boulder, Colorado 80305 USA; 2 University of Colorado, Boulder, Colorado 80305 USA

**Keywords:** anomaly boundary, boundary discontinuity, carrier phase, curve fitting, cycle slip, discontinuity (anomaly-BD), GPS, GPS jamming, precise point positioning (PPP), time transfer

## Abstract

The wide application of Global Positioning System (GPS) carrier-phase (CP) time transfer is limited by the problem of boundary discontinuity (BD). The discontinuity has two categories. One is “day boundary discontinuity,” which has been studied extensively and can be solved by multiple methods [1–8]. The other category of discontinuity, called “anomaly boundary discontinuity (anomaly-BD),” comes from a GPS data anomaly. The anomaly can be a data gap (i.e., missing data), a GPS measurement error (i.e., bad data), or a cycle slip. Initial study of the anomaly-BD shows that we can fix the discontinuity if the anomaly lasts no more than 20 min, using the polynomial curve-fitting strategy to repair the anomaly [9]. However, sometimes, the data anomaly lasts longer than 20 min. Thus, a better curve-fitting strategy is in need. Besides, a cycle slip, as another type of data anomaly, can occur and lead to an anomaly-BD. To solve these problems, this paper proposes a new strategy, i.e., the satellite-clock-aided curve fitting strategy with the function of cycle slip detection. Basically, this new strategy applies the satellite clock correction to the GPS data. After that, we do the polynomial curve fitting for the code and phase data, as before. Our study shows that the phase-data residual is only ~3 mm for all GPS satellites. The new strategy also detects and finds the number of cycle slips by searching the minimum curve-fitting residual. Extensive examples show that this new strategy enables us to repair up to a 40-min GPS data anomaly, regardless of whether the anomaly is due to a data gap, a cycle slip, or a combination of the two. We also find that interference of the GPS signal, known as “jamming”, can possibly lead to a time-transfer error, and that this new strategy can compensate for jamming outages. Thus, the new strategy can eliminate the impact of jamming on time transfer. As a whole, we greatly improve the robustness of the GPS CP time transfer.

## 1. Introduction

Global Positioning System (GPS) carrier-phase (CP) time transfer is a widely accepted high-precision time transfer method. This method provides much lower short-term noise than other time transfer methods, such as GPS code-based common-view (CV) time transfer and Two Way Satellite Time and Frequency Transfer (TWSTFT) [10–12]. However, discontinuities of up to 1 ns often occur in the CP time transfer. Whenever the carrier-phase processing re-initializes at a specific epoch, it needs to re-estimate the phase ambiguities based on the code measurements. The new phase-ambiguity estimation is often different from the previous estimation because of the code noise [1]. This leads to the discontinuity at this epoch. The discontinuity problem is an obstacle for the sophisticated time-transfer activities in many national timing laboratories.

Two cases lead to the re-initialization of the carrier-phase processing. One is when we process a new data batch. Of course, the carrier-phase processing needs to re-initialize and thus there is a jump at the beginning of the new data batch. We call this jump “data-batch boundary discontinuity.” This type of BD has been studied extensively and can be well solved by the “revised RINEX-Shift” algorithm [1–2] or other methods [3–8]. The other case is when we come across a GPS data anomaly. The anomaly can be missing data, bad data, or cycle slips. It is hard for the carrier-phase processing to keep the estimated parameters at the anomaly. Thus, it typically re-initializes after the anomaly. This type of discontinuity is called “anomaly boundary discontinuity (anomaly-BD)”. Since the anomaly occurs quite often at many national metrology institutes (e.g., three times in a month), the anomaly-BD has seriously impaired the robustness of the GPS CP time transfer. However, it receives little attention in the precise GPS timing community.

As an initial study of the anomaly-BD problem, we proposed a simple curve-fitting strategy for repairing the GPS data anomaly, in 2014 [9]. There, we did the 9th-order polynomial regression to fit the good data both before and after the anomaly. Then the fitted data during the time range of the anomaly is used to replace the anomaly. Statistically, the root mean square (RMS) of the curve-fitting residuals for phase measurements is typically smaller than 5 cm. And the RMS of the residuals for code measurements is typically ~ 0.6 m. We found out that this strategy can eliminate the anomaly-BD if the anomaly spans less than 20 min.

However, sometimes, the data anomaly lasts longer than 20 min. Thus, we want to find a better curve-fitting strategy and make it work for a longer term of data anomaly. Besides, cycle slips can also occur during the data anomaly. The cycle slip, as a type of data anomaly, can lead to an anomaly-BD. Thus, how to detect and remove the cycle slips is also a critical and practical problem in eliminating the anomaly-BD. This paper provides solutions to the above two problems.

Section 2 proposes the satellite-clock-aided curve fitting strategy with the function of cycle slip detection. This new strategy enables us to repair a longer term of GPS data anomaly. It also avoids the discontinuity coming from the cycle slips. To verify this new strategy, Sec. 3 provides extensive tests. As an example, to verify the time-transfer result using the repaired GPS data, we compare it with the time-transfer result via another GPS receiver at the same location. As another example, we compare the repaired GPS CP time transfer result with the TWOTFT (two way optical-fiber time and frequency transfer) result. These examples show the correctness of our new data-repairing strategy. Section 4 discusses an important application of the new strategy. There, we find that a GPS jamming event can lead to an anomaly-BD in GPS carrier-phase time transfer. The new strategy successfully eliminates the anomaly-BD due to jamming. Section 5 concludes this paper.

## 2. New GPS-Data-Repairing Strategy

In 2014, we found that the residual of the 9^th^-order polynomial regression for the phase data can be as small as 6 mm, in terms of root-mean-square (RMS), for Block IIF GPS satellites. However, for non-IIF satellites, the RMS of the residual became ~3 cm, which was approximately five times greater than that for Block IIF GPS satellites. This is because the non-IIF satellite clocks are noisier than the IIF satellite for a short time period (< 3 hours). The satellite clock noise fundamentally limits the performance of curve fitting. Thus, we can only repair the GPS data anomaly of up to 20 min, at that time.

To achieve better curve fitting, here, we design the “satellite-clock-aided curve fitting” strategy which applies the satellite clock correction. First, we extract the high-precision satellite clock information from the International GNSS Service (IGS) clock file. Then we apply the satellite clock correction to both code and phase data, for each GPS satellite. Thus, we get the “updated” code and phase data. After that, we do polynomial curve fitting for the updated code and phase data. The RMS of the residuals for the updated code data should be almost the same as that using the old strategy, since the satellite clock noise is not a major contributor to the code noise. However, the RMS of the residuals for the updated phase data should become much smaller than that using the old strategy, because the satellite clock noise does contribute a lot to the phase noise. In the end, we get the “final fitting results” by removing the satellite clock correction from the above code and phase fitting results. If a data anomaly occurs, we use the “final fitting results” to replace the original code and phase data during the anomaly.

**Fig. 1:**
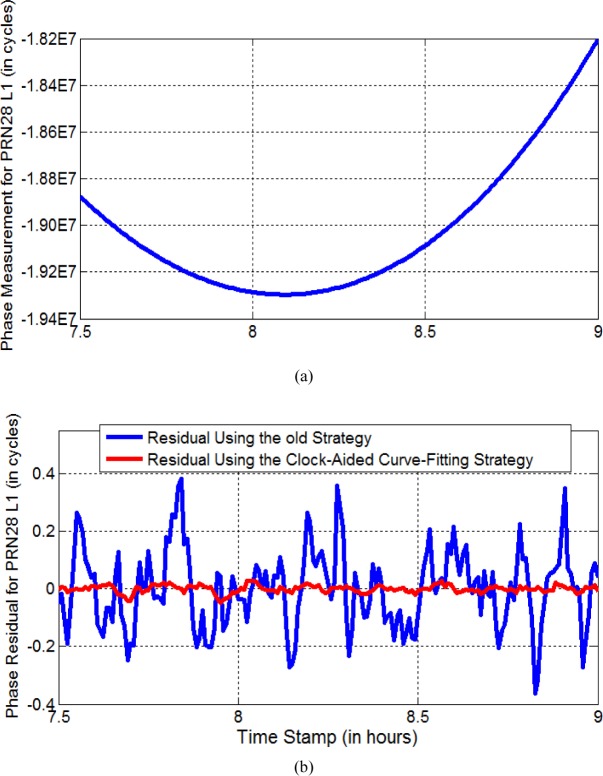
Phase residuals for PRN28 L1, using the old strategy (blue curve in (b)) and the satellite-clock-aided curve-fitting strategy (red curve in (b)). The top plot (a) shows the phase data for PRN28 L1. Note, the phase data were recorded by the “*NISA*” receiver on MJD 56325.

To confirm that the phase residual using the “satellite-clock-aided curve fitting” strategy does become much smaller than that using the old strategy, we study the phase data for the satellite designated as PRN 28, recorded by a geodetic GPS receiver, from 7:30:00 to 9:00:00 on Modified Julian Date (MJD) 56325. This GPS receiver, named “*NISA*”, is located at NIST (National Institute of Standards and Technology). The reference time for PRN 28 is generated by a Block IIR rubidium clock. Figure 1(a) shows the phase data. The blue curve in Fig. 1(b) shows the phase residual using the old strategy. Statistically, the RMS of phase residual is approximately 0.14 cycle, corresponding to 2.7 cm. This is very close to our previous result (i.e., ~ 3 cm) (see Fig. 4 in [9]). The red curve in Fig. 1(b) shows the phase residual using the “satellite-clock-aided curve fitting” strategy. Clearly, the phase residual becomes significantly smaller than that using the old strategy. The RMS of the phase residuals is only 0.013 cycle, i.e., 2.5 mm. This demonstrates that the “satellite-clock-aided curve fitting” strategy is much better than the old strategy for non-IIF satellites. Even for the IIF satellites, we can still get some improvement. Remember that the old strategy gives ~ 6 mm phase residual [9], while this “satellite-clock-aided curve fitting” strategy can easily reach ~ 3 mm phase residual.

Now that we have reached a very good result of fitting missing data and bad data, we want to study the cycle slips in the phase measurement. As mentioned in Sec. 1, a cycle slip is another reason that leads to an anomaly-BD. To detect a cycle slip, we do the forward detection, which does the polynomial curve fitting for a number of data points (e.g., 60 points) and then check the difference between the extrapolated value and the actual value at the 61^st^ point. If the difference is above a pre-defined threshold (e.g., two cycles), then we know that there is a cycle slip. To estimate the value of this cycle slip, we perform “two-end detection.” This consists of fitting a polynomial curve to the data set with the cycle-slip data point as the middle point (e.g., the data set has 121 points and the cycle-slip point is the 61^st^ point). We adjust those phase-measurement values after the cycle-slip point by an integer. After a series of adjustments of the integer, we find the integer that results in the minimal RMS of the residuals (typically, the minimal RMS is less than 2 cm). Of course, the satellite clock correction is already applied before we do the curve fitting in all of the above operations. Our tests show that these procedures work for most cases. If the minimal RMS is greater than 2 cm, then we do not apply the cycle slip correction (that means, we do not repair the cycle slips), because the repair is not good enough.

**Fig. 2:**
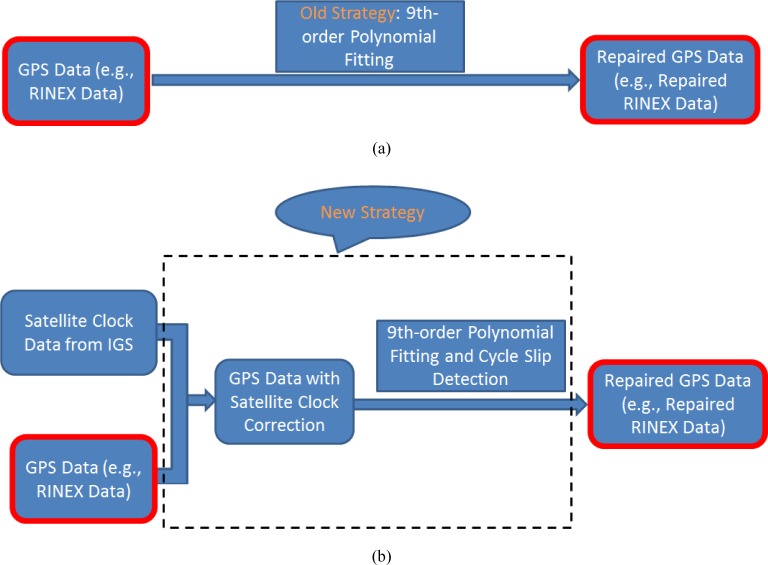
Illustrations of the old strategy (a) and the new strategy (b).

Here, we call the “satellite-clock-aided curve fitting” strategy with the function of cycle slip detection, the “new” strategy, for the sake of simplicity. Figure 2 illustrates the structural difference between the old strategy and the new strategy.

## 3. Verification of the New Strategy

As discussed in Sec. 2, the new strategy not only provides excellent curve-fitting result (~3 mm in RMS), but also removes the cycle slips. This section describes how its performance has been tested in extensive scenarios. Generally, the case of missing data occurs much more frequently than the case of bad data. Thus, many scenarios here are about missing data. A case of missing data together with cycle slips is also studied to verify the cycle slip repairing mechanism in the new strategy. Some comparisons, such as a comparison with another GPS receiver and a comparison with TWOTFT, are introduced to further confirm the new strategy.

**Fig. 3:**
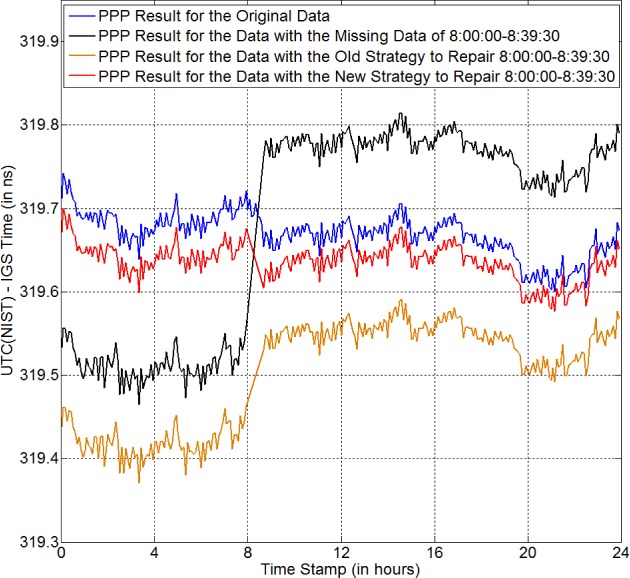
PPP results using the new strategy (red curve) and the old strategy (orange curve), for the case of 40 min missing data. The blue curve is the PPP result for the original GPS data. The black curve is the PPP result for the same data but missing the data from 8:00:00 to 8:39:30.

The first example we give is an “artificial” missing data. This is a good way to verify the correctness of a data-repairing strategy, because we already know the correct result. *NISA*, a GPS receiver at NIST, is used to record the GPS data every 30 s on MJD 56325. The data are continuous and no data are missing. The reference time for *NISA* is UTC(NIST) with a constant delay. We use the NRCan Precise Point Positioning (PPP) software package to do the CP time transfer processing [13]. The PPP result provides the time difference between the local time (i.e., UTC(NIST) in this case) and the IGS time. The PPP result for the GPS data is shown by the blue curve in Fig. 3. Clearly, the result is continuous since there is no data anomaly. Now, we “make” an anomaly by deleting the data from 8:00:00 to 8:39:30 (39.5 min). The PPP result is shown by the black curve in Fig. 3. Obviously, there is an anomaly-BD of ~250 ps. This indicates that an anomaly can invalidate the time-transfer result, since we need to re-estimate the phase ambiguities after the anomaly. Next, we do curve-fitting to repair this anomaly. First, we use the old strategy to get the repaired GPS data. Then we run PPP for the repaired GPS data and get the PPP result as shown by the orange curve. We can see that there is still a discontinuity in the orange curve. Besides, the whole curve is shifted down by about 100 ps to 250 ps from the original curve (i.e., blue curve). These indicate that the old strategy does not work well for the case of a 40-min data anomaly. In Ref. [9] (Fig. 6), we observed a result similar to the orange curve shown here. Next, we use the new strategy to repair the 40-min missing data (red curve in Fig. 3). Obviously, the red curve follows the blue curve very well. The time change from 7:55:00 to 8:40:00 in the blue curve is −67 ps, while the time change from 7:55:00 to 8:40:00 in the red curve is −71 ps. Thus, the result of the new strategy at the anomaly has the same trend as the original result. The whole red curve is about −30 ps away from the blue curve. This may come from the imperfect fitting for the code data. As a few papers mentioned [1, 6, 8], code data are used to determine the absolute time. The code data are much noisier than the phase data, which makes the residuals of code fitting large (~ 60 cm). In practice, tens of picoseconds offset does not matter. The accuracy of carrier-phase time transfer is at the level of 100 ps. Thus, tens of ps offset can be neglected. From the above discussion, we know that the new strategy can repair the data properly and the time-transfer result using this new strategy is close to the true result.

**Fig. 4:**
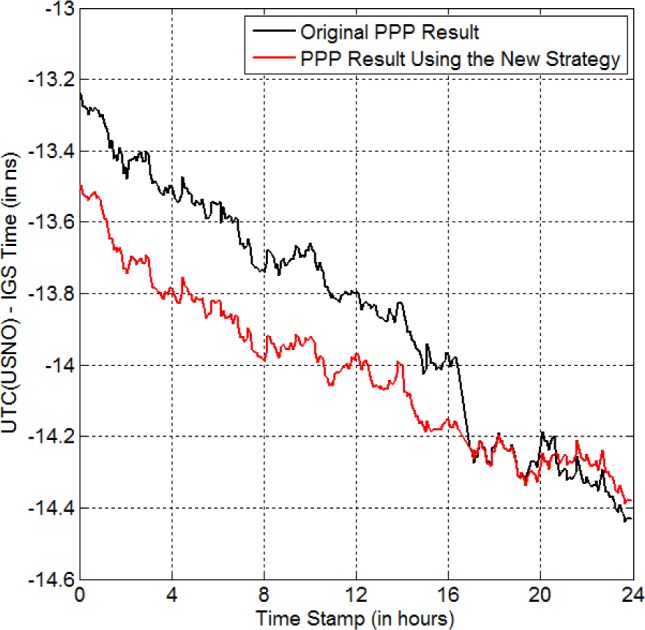
New strategy for eliminating the anomaly-BD in UTC(USNO) on MJD 57113 (red curve). The black curve is the PPP result for the original GPS data. There is no data from 16:30:00 to 16:52:30, in the original data.

*USN7* is a GPS receiver located at the United States Naval Observatory (USNO). Its reference time is UTC(USNO) with a constant delay. We find that there is no data recorded by this receiver from 16:30:00 to 16:52:30 (i.e., 22.5 min. Note, the GPS data were recorded always every 30 s throughout this paper) on MJD 57113. Thus, there is an anomaly in the original data. The PPP result without any data repair is shown by the black curve in Fig. 4. We can see that there is a jump of −223 ps at the anomaly. If we repair this anomaly using the new strategy, we get the red curve. Clearly, the jump disappears and the whole time-transfer result becomes reasonable.

**Fig. 5:**
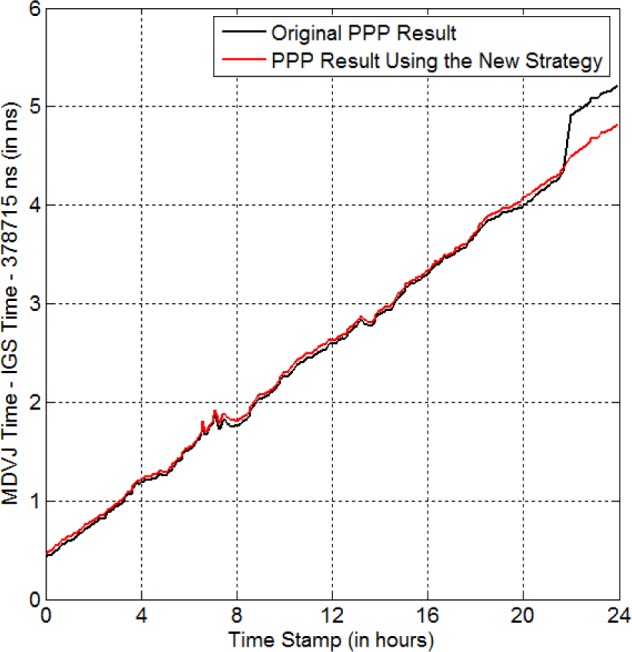
New strategy for eliminating the anomaly-BD in the *MDVJ* time on MJD 56884 (red curve). The black curve is the PPP result for the original GPS data. There is no data from 21:41:30 to 21:59:30, in the original data.

As another example, *MDVJ* is a GPS receiver in Mendeleevo, Russia. Its reference time is a Hydrogen maser. We call this reference time the “*MDVJ* time”. We notice that there is no GPS data from 21:41:30 to 21:59:30 (i.e., 18.0 min) on MJD 56884. The black curve in Fig. 5 shows the time-transfer result for the original data. Obviously, there is a jump of +574 ps. The red curve in Fig. 5 is the time-transfer result using the new strategy. The jump no longer exists and the whole red curve becomes smooth.

**Fig. 6:**
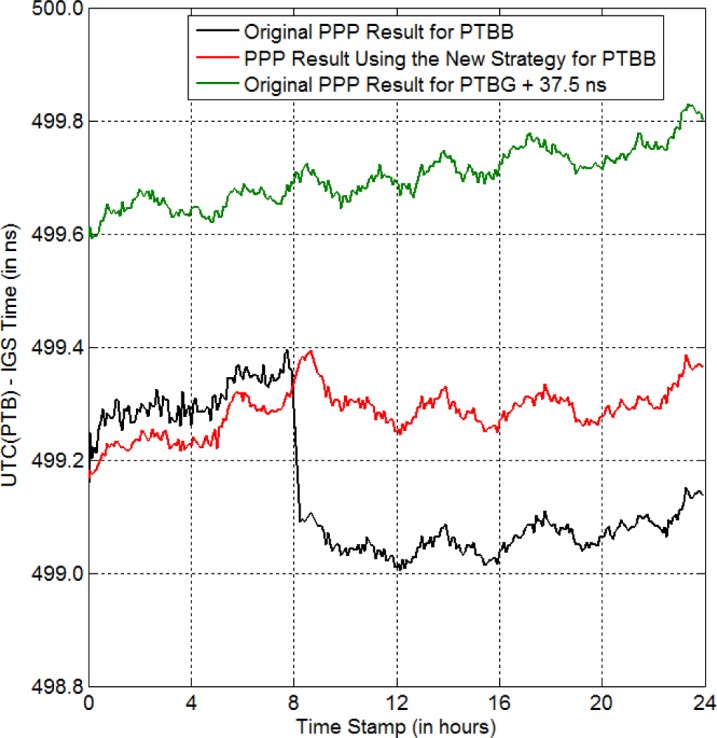
New strategy for eliminating the anomaly-BD in UTC(PTB) on MJD 56901 (red curve). The black curve is the PPP result for the original *PTBB* GPS data. There is no data from 8:00:00 to 8:14:00, in the original *PTBB* data. The PPP result for *PTBG* is provided as a reference (green curve).

Similar results of successfully eliminating the anomaly-BD as shown in Figs. 4–5 can be listed as many as we want. All these demonstrate the correctness of the new strategy. To further confirm the new strategy, we compare the time-transfer results using two different GPS receivers at the same location. *PTBB* and *PTBG* are two GPS receivers at PTB (Physikalisch-Technische Bundesanstalt), Germany. UTC(PTB) is the reference time for both receivers. In theory, the time-transfer result via *PTBB* should be the same as that via *PTBG*, because they have a common clock. In practice, there could still have some small (< 100 ps) time-transfer difference between the receivers due to noise and equipment delays. However, generally speaking, the difference within one day is so small that we can assume that *PTBB* and *PTBG* are equivalent performers. On MJD 56901, we have no data from 8:00:00 to 8:14:00 (i.e., 14.0 min) for *PTBB*. Luckily, we have data for the same period for *PTBG*. Thus, this is a good test for the new strategy, since we can get the “right” answer from *PTBG*. From the black curve in Fig. 6, we can see that there is a jump of −267 ps due to the missing data of *PTBB*. Now, similar to what we have done previously, we use the new strategy to repair the missing data and run PPP again. The result is shown by the red curve. The jump disappears. Most importantly, the red curve follows the trend of the “right” answer (i.e., the green curve) quite well. In contrast, the black curve is seriously distorted due to the missing data. This indicates that the new strategy maintains the validity of the time-transfer result. If there is a data anomaly and we do not repair it, the time-transfer result contains errors that is not indicative of the performance of the clocks we are measuring.

The above examples have demonstrated the power of the new strategy for repairing missing data. As we mentioned in Sec. 2, cycle slips occur from time to time. Using the procedures in Sec. 2, it is not hard to repair the cycle slips at a specific epoch, if we have good GPS data around this epoch. However, if a cycle slip occurs immediately after the missing data, then the situation becomes more complicated. We should mention that this scenario can be common. For example, if a GPS receiver stops working and re-starts later, then it will re-count the carrier-wave cycles, creating an instance where we have both missing data and cycle slips. Thus, this is a good test for the cycle-slip-detection mechanism.

**Fig. 7:**
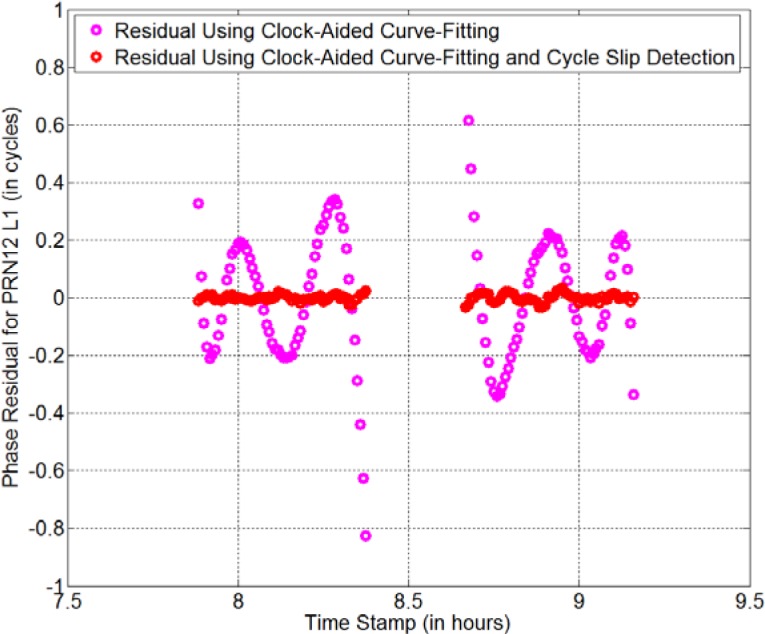
Verification of cycle slip detection (red curve). The red curve is the result using the new strategy, which does both satellite-clock-aided curve fitting and cycle slip detection. The RMS of residual after removing slips is only 2.5 mm (red curve). If cycle slip is not removed, the RMS of residual is 4.4 cm (magenta curve).

**Fig. 8:**
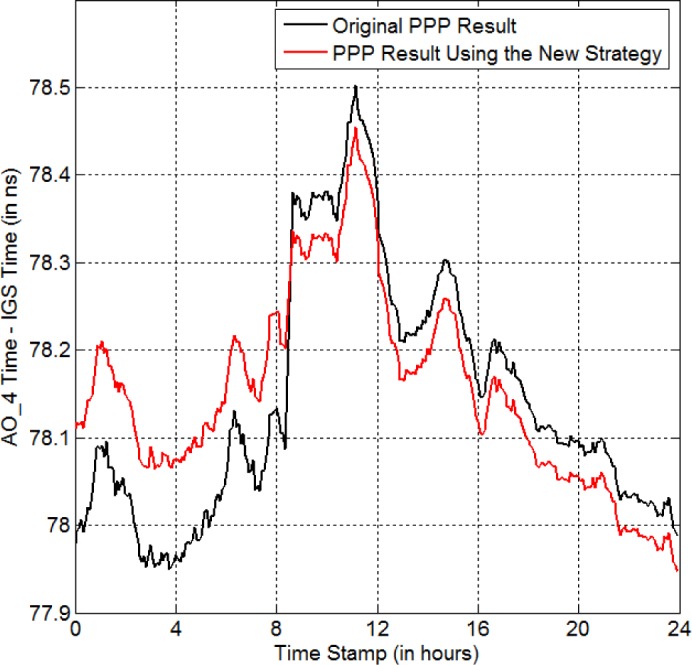
New strategy for eliminating the anomaly-BD in the *AO_4* time on MJD 56909 (red curve). The black curve is the PPP result for the original GPS data. There is no data during 8:23:00–8:39:30, in the original data.

As an example, we find that there is no GPS data from 8:23:00 to 8:39:30 for the *AO_4* receiver on MJD 56909. *AO_4* is located at AOS (Astrogeodynamical Observatory Space Research Centre) in Poland. Careful investigation shows that there are cycle slips for several satellites during the missing-data period. If we only use the satellite-clock-aided polynomial fitting to repair the missing data, the residual is as large as 0.23 cycle = 4.4 cm for PRN12 on the L1 carrier (see the magenta curve in Fig. 7). Actually, the residual is oscillating and becomes even larger near the missing-data period (> 0.6 cycle). Thus, we can imagine that the curve-fitting error during the missing-data period should be greater than 0.6 cycle. This big fitting error is not acceptable for eliminating the anomaly-BD. As discussed in Sec. 2, we can reach the residual of ~ 3 mm if there is only missing/bad data. For such a big residual, the program automatically initiates the cycle-slip detection/repair procedures. We find that if we adjust the phase data after the missing-data period by −15 cycles, the residual becomes as small as 0.013 cycle = 2.5 mm (see the red curve in Fig. 7). Thus, the cycle slip number is −15 cycles for this case. We do the same thing for all other observable satellites (both L1 and L2). The time-transfer result for the repaired GPS data is shown by the red curve in Fig. 8. The black curve is the PPP result for the original GPS data without any repair. Clearly, the new strategy removes the jump of +293 ps in the black curve successfully. This indicates that our cycle-slip-detection procedures work properly.

**Fig. 9:**
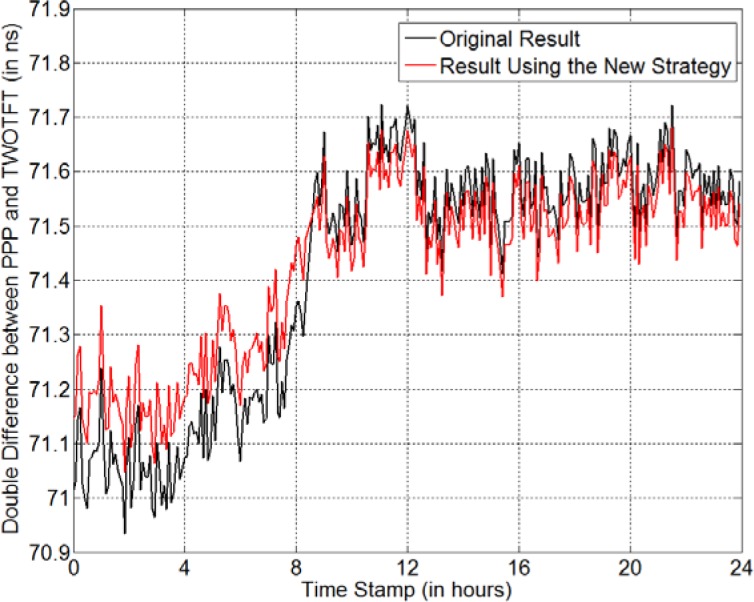
Double difference between PPP and TWOTFT. The black curve is the double difference when no GPS data are repaired. The red curve is the double difference when GPS data are repaired by the new strategy.

Next, we use TWOTFT to further verify that the new strategy does properly repair the *AO_4* GPS data on MJD 56909. As a terrestrial time-transfer technique, TWOTFT is almost completely independent from GPS time transfer. Thus, a comparison between GPS CP time transfer and TWOTFT can be used to verify the correctness of the GPS CP time-transfer result. There is a two-way optical fiber link between AOS and PL (Polish Atomic Time Scale) [14]. The physical distance between the two laboratories is 268 km. *AO_4* and *GUM4* are the two GPS receivers, at AOS and PL, respectively. The time references for the optical fiber link and the GPS receivers are the same at each station. Thus, a double difference between GPS CP time transfer and TWOTFT reveals how well GPS matches TWOTFT. If we do not repair the missing *AO_4* GPS data from 8:23:00 to 8:39:30 on MJD 56909, the result of the double difference shows an obvious jump of +218 ps at the anomaly (black curve in Fig. 9). In contrast, there is no jump at the anomaly, if the new strategy is applied to repair the *AO_4* GPS data as described in the previous paragraph (red curve in Fig. 9). Note, the time change during the missing data period in the red curve is only +59 ps, which should not be considered as a jump. Also, we notice that the GPS CP time transfer varies from TWOTFT by ~ 450 ps after 24 hours if no data repair is done (black curve). On contrary, it varies by only ~300 ps if we repair the data (red curve). This indicates that the red curve is more correct than the black curve. Thus, the new data-repairing strategy does repair the GPS data properly and the time-transfer result using the repaired data is closer to the true value.

In summary, this section has provided many examples showing that the new strategy can repair any missing/bad data and cycle slips, if the anomaly lasts no longer than 40 min. This leads to the question of whether we can repair an even longer anomaly. The answer is that if frequency transfer is the main concern, we can do a longer repair (e.g., 70 min). However, if we need to do time transfer (that is, we need to know the absolute time), then 40 min is probably the upper limit, to be conservative. This is because the absolute time comes from the code, and the curve-fitting for the code is not very good because of the code noise. The error in the curve fitting for the code becomes larger as the data gap becomes longer. Another important reminder is that the curve-fitting strategies (both old and new) only work well for national laboratories that have near-perfect reference clocks, or those GPS receivers with very good rubidium/cesium/H-Maser clocks as their references. For a non-precise clock (such as a quartz oscillator), the curve fitting could be tens of centimeters or even more away. Therefore, PPP still needs to re-estimate the phase ambiguities after the anomaly and thus the anomaly-BD cannot be removed.

## 4. Applications

From the above analysis, we know that the new strategy can repair any GPS data anomaly, if the anomaly spans no longer than 40 min. GPS jamming can block the reception of GPS satellites so that no data is recorded. Thus, a jamming event causes a GPS data anomaly. An important application of the new strategy is that it enables us to repair the anomaly due to jamming and thus eliminates the impact of GPS jamming on time transfer.

Although the impact of GPS jamming on real-time positioning has been discussed for many years [15–18], the impact of jamming on time transfer has received little or no attention in the literature. To study this issue, a commercial GPS jamming detector has been installed at NIST since May 2014. We find that most jamming events do not lead to any obvious positioning/timing errors, probably because of the advanced anti-jamming techniques already implemented in the GPS receiver. However, this is not always true. In fact, sometimes, we observe a jump (i.e., anomaly-BD) in GPS carrier-phase time transfer, because of jamming.

**Fig. 10:**
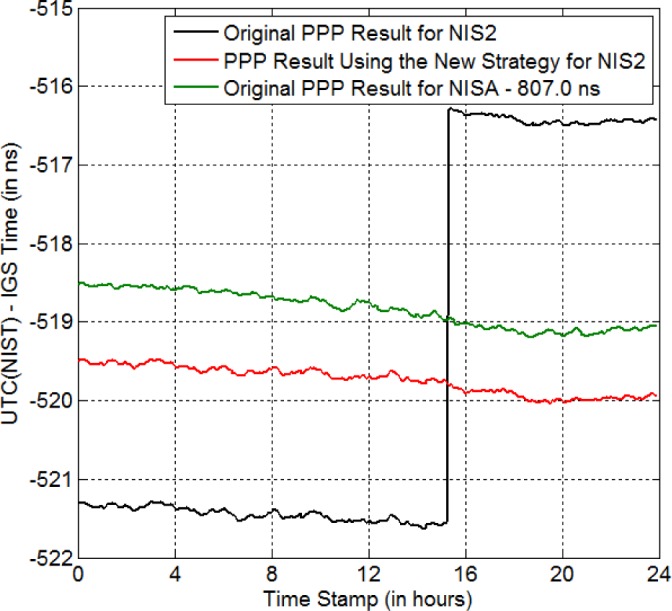
New strategy for eliminating the anomaly-BD due to jamming. The jamming event occurs at around 15:18:00 on MJD 57048. The black curve is the PPP result for the original *NIS2* GPS data. The red curve is the PPP result for the repaired *NIS2* GPS data. The PPP result for *NISA* is provided as a reference (green curve).

As an example, the jamming detector detected a jamming event lasting 43 s, at around 15:18:00 on Jan. 26, 2015 (i.e., MJD 57048). Because of the jamming, *NIS2*, a GPS receiver at NIST, only received the GPS signal from two satellites at 15:18:30. If we process the GPS data using PPP, we get the black curve shown in Fig. 10. Clearly, there is a jump of as large as ~ 5 ns when the jamming event occurs! This large jump ruins the time-transfer result.

We should mention that the *NIS2* receiver is close to Broadway, a main street in Boulder, CO. The jamming source may have come from a car passing by. Another GPS receiver *NISA*, also located at NIST, has the same reference time as *NIS2*, i.e., UTC(NIST). This receiver is further away from Broadway and thus was not affected by the jamming event on MJD 57048, successfully receiving nine satellites at 15:18:30. Thus, the time-transfer result using *NISA* represents the true value. The green curve in Fig. 10 shows the PPP result for *NISA*, and no time jump is visible at ~15:18:00. Compared to the black curve, we can see how wrong the time-transfer result using *NIS2* is.

Now we know that a jamming event can lead to the scenario of missing data, and thus invalidate the GPS carrier-phase time transfer result. The next question is that what we shall do when a jamming event occurs. As a post-processing technique, our solution is to repair the GPS data at jamming using the new strategy. Here, we repair the *NIS2*’s GPS data at ~15:18:30 using the new strategy and then run PPP for the repaired data. The result is shown by the red curve in Fig. 10. Obviously, the large jump of around 5 ns disappears. Besides, the red curve has the same trend as the green curve. Since the green curve represents the “right” answer, this indicates that the PPP result using the new strategy is closer to the true value.

The above example demonstrates how powerful the new strategy is when compensating for jamming events that last 40 min or less. Admittedly, if the jamming lasts for longer than 40 min, the new strategy does not work well. However, this long-term jamming event is rare. If it does occur, that is the time we have to find out the jamming source.

## 5. Conclusions

To conclude, from this paper, we know that a few minutes of GPS data anomaly can lead to an anomaly-BD of more than 200 ps. This paper has proposed a new GPS-data-repairing strategy to eliminate the anomaly-BD. Extensive tests have shown that the new strategy successfully removes the anomaly-BD when the GPS data anomaly lasts 40 min or less. This same strategy can also compensate for the loss of data due to jamming. Thanks to this strategy, we have improved the robustness of GPS CP time transfer, making it possible to measure the performance of remote Cs fountain clocks even when there are GPS data anomalies.
